# SeSBAT: Single Subject Brain Analysis Toolbox. Application to Huntington’s Disease as a Preliminary Study

**DOI:** 10.3389/fnsys.2020.488652

**Published:** 2020-09-29

**Authors:** Alicia Palomar-Garcia, Estela Camara

**Affiliations:** ^1^Cognition and Brain Plasticity Unit, IDIBELL (Institut d’Investigació Biomèdica de Bellvitge), Barcelona, Spain; ^2^Department of Cognition, Development and Educational Psychology, University of Barcelona, Barcelona, Spain

**Keywords:** Huntington’s disease, individual differences, neurodegeneration, neuroimaging, structural MRI

## Abstract

Magnetic resonance imaging (MRI) biomarkers require complex processing routines that are time-consuming and labor-intensive for clinical users. The Single Subject Brain Analysis Toolbox (SeSBAT) is a fully automated MATLAB toolbox with a graphical user interface (GUI) that offers standardized and optimized protocols for the pre-processing and analysis of anatomical MRI data at the single-subject level. In this study, the two-fold strategy provided by SeSBAT is illustrated through its application on a cohort of 42 patients with Huntington’s disease (HD), in pre-manifest and early manifest stages, as a suitable model of neurodegenerative processes. On the one hand, hypothesis-driven analysis can be used to extract biomarkers of neurodegeneration in specific brain regions of interest (ROI-based analysis). On the other hand, an exploratory voxel-based morphometry (VBM) approach can detect volume changes due to neurodegeneration throughout the whole brain (whole-brain analysis). That illustration reveals the potential of SeSBAT in providing potential prognostic biomarkers in neurodegenerative processes in clinics, which could be critical to overcoming the limitations of current qualitative evaluation strategies, and thus improve the diagnosis and monitoring of neurodegenerative disorders. Furthermore, the importance of the availability of tools for characterization at the single-subject level has been emphasized, as there is high interindividual variability in the pattern of neurodegeneration. Thus, tools like SeSBAT could pave the way towards more effective and personalized medicine.

## Introduction

Magnetic resonance imaging (MRI) is becoming increasingly important as an excellent non-invasive tool for the quantification of brain anatomy and function in a more sensitive, reliable, and illustrative manner than commonly employed techniques. Clinical neuroradiologists typically rely on visual inspection for MRI data interpretation. Although specific atrophy patterns have been identified in various neurodegenerative disorders, the power of visual inspection is intrinsically limited for their diagnosis and monitoring, as it only allows for a qualitative description of the anatomy, and certain signs of neurodegeneration are not visible until the damage is significantly advanced. For this reason, it would be necessary to make a shift in the radiological paradigm towards the use of quantitative imaging, a crucial step towards improved disease detection and diagnosis. Various recent studies using quantitative imaging have revealed abnormalities in gray (GM) and white matter (WM) integrity in different neurodegenerative disorders such as Alzheimer’s disease (AD; Pini et al., 2016; Chandra et al., 2019; Kunst et al., 2019), Huntington’s disease (HD; Tabrizi et al., 2012; Aylward et al., 2013; Minkova et al., 2018), amyotrophic lateral sclerosis (ALS; Rajagopalan et al., 2013; Ferraro et al., 2017) and Parkinson’s disease (PD; Shao et al., 2015; Kunst et al., 2019). Furthermore, the characterization of pre-symptomatic MRI biomarkers of neurodegeneration would pave the way for the design of more sensitive protocols for clinical diagnosis by highlighting the earliest affected brain regions (Schuster et al., 2015). Thus, quantitative MRI may bring diagnosis back to the pre-symptomatic stage or early symptomatic stages, allowing early recruitment into clinical trials for seeking effective disease-modifying treatments. Furthermore, the early detection of possible anatomical markers of neurodegeneration would have the potential to alert neuroradiologists to act in an optimal window, geared towards precision and preventive medicine.

While quantitative imaging techniques are currently used in research, mainly in the field of cognitive neuroscience, they have not been applied to clinical routine. This is mainly due to the complex processing routines required for the extraction of MRI biomarkers, such as the parcellation of the brain into cortical and subcortical regions, which are laborious and time-consuming for clinical practitioners. To address these limitations various standardized and optimized protocols should be integrated with a user-friendly interface to provide a more straightforward, handy, and automatic approach for the processing of anatomical MRI data in clinics. Moreover, commonly used MRI pulse sequences and analysis pipelines in neurodegenerative disorders tend to be similar (Bede, 2017).

Until now, most research studies on neurodegenerative disorders using neuroimaging approaches have typically compared two groups, regarding individual differences as a source of noise often eliminated by averaging data across participants (Kanai and Rees, 2011). However, different anatomical biomarkers allow for the detection of more subtle differences and the characterization of neurodegeneration at the single-subject level (Garcia-Gorro et al., 2019). Single-subject studies provide data based on the individual subject, which will be more accurate for clinical prognosis than data resulting from averaging a group of individuals. Understanding the neurobiological basis of individual variability is of utmost importance to developing specific biomarkers and identifying possible subgroups of patients for successful targeted clinical trials (Garcia-Gorro et al., 2019).

All that together leads to the use of common imaging approaches for seemingly different conditions. This has motivated the development of the Single Subject Brain Analysis Toolbox (SeSBAT). This toolbox represents a user-friendly approach that integrates standard pipelines for the processing of anatomical neuroimaging data at a single-subject level to detect potential prognostic factors that could be used for predicting an increased risk of developing a particular neurodegenerative disorder.

Different automated segmentation software packages and approaches have been developed to identify brain morphological changes in the brain. Some tend to overestimate the volume of the regions extracted while others tend to underestimate it, and for specific tasks, other approaches might provide more accurate and reliable results (Kassubek et al., 2011; Eggert et al., 2012; Johnson et al., 2017). All of them are subject to inaccuracies in the segmentation of certain regions (Johnson et al., 2017; Xie et al., 2019). Currently, no method is considered superior in all aspects.

From the different possibilities, SeSBAT integrates both Freesurfer and SPM software, two of the more popular approaches for brain morphometry. Although both packages provide detailed segmentation of the gray matter (GM) in the brain, they do it in two complementary different ways. On the one hand, Freesurfer provides a detailed parcellation of the cortex and subcortical regions by combining volumetric and surface-based segmentation, using a template-driven approach (Fischl, 2012). On the other hand, SPM implements a voxel-based morphometry (VBM) approach based on both linear and non-linear registration of the brain to a standardized template that allows to segment brain tissues by assigning tissue probabilities per voxel (Ashburner and Friston, 2005).

This article describes the functionality of SeSBAT and its application to HD, which is a devastating neurodegenerative genetic disorder that shares many features with other more common neurodegenerative disorders such as AD and PD, but it is distinct in that individuals at risk of developing this disease can be identified by predictive genetic testing before clinical manifest onset (Ross and Tabrizi, 2011). This makes HD a suitable model for testing SeSBAT in the early stages of neurodegeneration. In other neurodegenerative diseases, specific diagnostic tests are not available before the appearance of first symptoms, a point in which neural degeneration is generally advanced. The early stages of neurodegeneration are generally asymptomatic, and, therefore, difficult to identify in advance.

Neuroimaging studies in HD have shown that it is possible to detect changes in early HD patients. Although the neurodegeneration is extended throughout the brain (Tabrizi et al., 2009, 2012), the most robust finding so far has been the caudate atrophy. In particular, atrophy in striatal structures has systematically been observed, more prominently in dorsal caudate and putamen, starting in the head of caudate in preclinical individuals (Rosas et al., 2003; Kassubek et al., 2004). Indeed, converging findings indicate that the striatum is the earliest region to show volume loss (Aylward et al., 2004; Tabrizi et al., 2009, 2011; Nopoulos et al., 2010). Changes in the caudate and the putamen have been detectable more than 15 years before expected disease onset (Paulsen et al., 2010). Moreover, significant volume reduction in the basal ganglia can be identified over 12 months in both pre-symptomatic and manifest HD when compared to controls (Majid et al., 2011; Tabrizi et al., 2011). Besides, the striatal neuronal loss is associated with CAG repeat length (Penney et al., 1997; Rosas et al., 2001; Kassubek et al., 2004), and also with motor and cognitive dysfunction (Montoya et al., 2006; Guo et al., 2012). Altogether highlights the important role of the striatum as a biomarker in neuroimaging for monitoring disease progression in HD (Harris et al., 1999; Aylward et al., 2004; Paulsen et al., 2008; Hobbs et al., 2010).

In this work, we hence do not propose novel biomarkers for neurodegeneration but we present a new tool that integrates widely used research techniques to analyze anatomical brain imaging data embedded in a user-friendly interface, to bridge one of the gaps between research and clinical practice.

## Overview of SeSBAT Design

**Figure 1:**
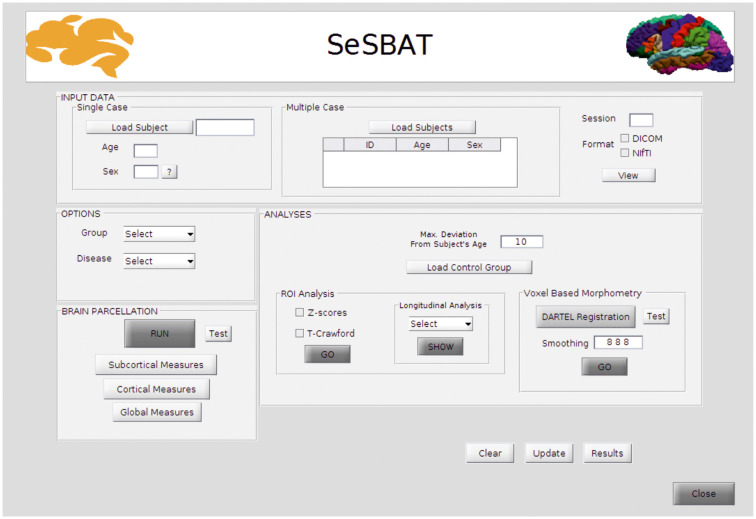
Main graphical user interface (GUI) of SeSBAT.

SeSBAT was developed in a MATLAB^®^ environment that provides a graphical user interface (GUI) framework (Figure 1), allowing users-toolbox interaction without the need for prior programming knowledge. This toolbox combines and integrates different freely available neuroimaging software packages such as FreeSurfer^1^ and SPM^2^ to provide automated, standardized, and optimized data processing of imaging data. SeSBAT is freely available upon request at http://brainvitge.org/.

### Modules Included in SeSBAT

#### Input Data

Data can be loaded for individual subjects or for a group of multiple subjects that will be analyzed independently. The toolbox is compatible with DICOM and NIfTI data formats. However, when loaded, DICOM images will be internally converted into NIfTI for posterior processing. Once the conversion is done, there is an option for visualizing the converted data, as the FreeSurfer command, *Freeview*^1^, has been integrated with SeSBAT.

#### Options

First, the group is selected to identify the participant as a patient or control individual. Then, the disease is selected to predefine a set of brain regions as the target of ROI-based analysis as per the disease under study.

**Figure 2:**
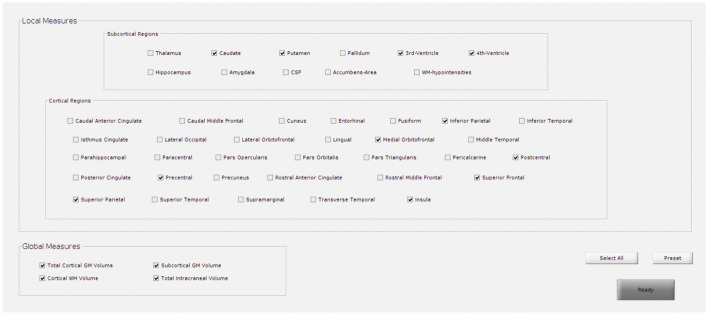
GUI for the selection of regions to target in regions of interest (ROI)-based analysis. This example shows the predefined regions for Huntington’s disease (HD).

Reducing the number of regions to analyze helps avoid the problems of multiple comparisons. If HD is selected, for instance, the predefined subcortical regions will be those reported as potential hallmarks of disease progression in the major cross-sectional and longitudinal structural MRI studies in pre-HD and early symptomatic HD patients (see review Weir et al., 2011), which are: the caudate, the putamen, the accumbens area, and the ventricles. Besides, some cortical areas (precentral, postcentral, superior frontal, inferior and superior parietal, insula, and medial orbitofrontal), and global measures will also be included (Figure 2).

#### Brain Parcellation

In this section, the brain is automatically parcellated into different subcortical and cortical regions. Specifically, cortical volume and thickness measures are estimated using Freesurfer^1^. From this parcellation, it is possible to extract biomarkers that have previously been related to neurodegeneration: subcortical volume, cortical volume, surface area, thickness and curvature, some global measures of GM and WM volumes, as well as the total intracranial volume (TIV). TIV is used to normalize the above-mentioned measures and adjust for head size differences.

**Figure 3:**
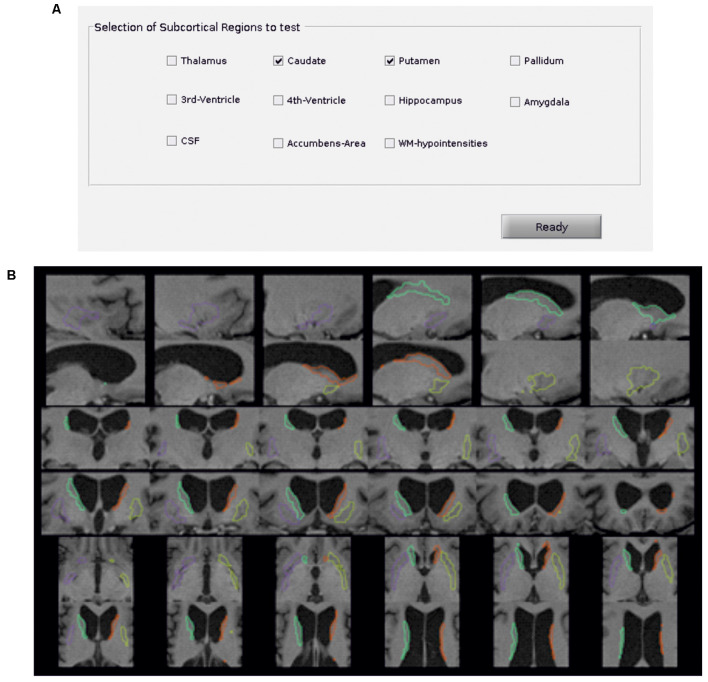
Subcortical parcellation quality control. **(A)** Selection panel to define the subcortical regions to test. In the example, the caudate and putamen are the selected ROIs. **(B)** Visualization is generated by the package *visualqc* to test subcortical parcellation. The resulting parcellation of left (green) and right (orange) caudate and left (purple) and right (yellow) putamen is shown at increasing levels of zoom.

**Figure 4:**
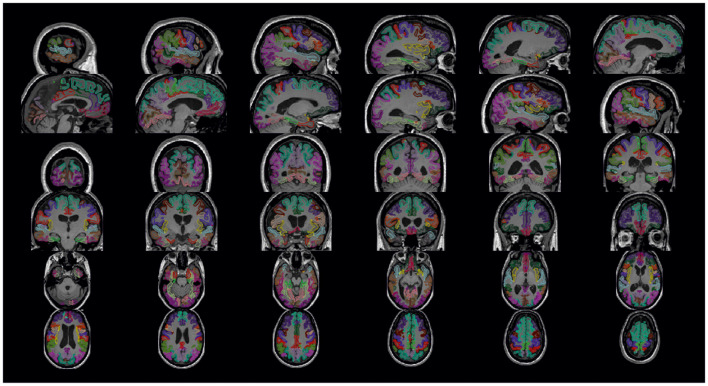
Cortical parcellation quality control. Visualization is generated by the package *visualqc* with sagittal, coronal, and axial views of the resulting cortical parcellation.

Additionally, the Python package *visualqc* (Raamana, 2018) has been integrated to assist neuroradiologists in testing the quality of brain parcellation. When testing subcortical parcellation (Figure 3), the user will be asked to select the target regions for testing (Figure 3A). Then, the software provides a figure containing the structural delineation of the previously selected subcortical regions (Figure 3B). A similar figure is provided for the whole brain when testing cortical parcellation (Figure 4).

#### Analyses

**Figure 5:**
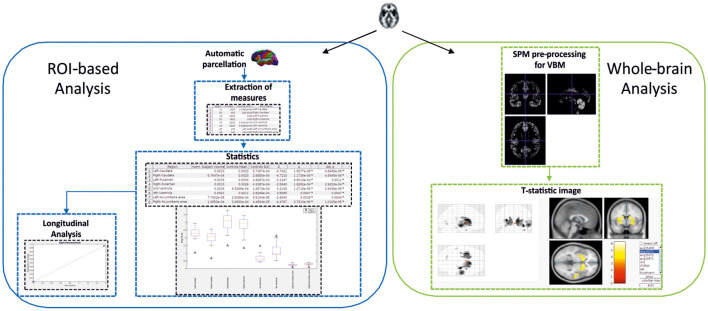
Schema showing the two-fold strategy provided by SeSBAT. The ROI-based analysis (left) starts with the automatic parcellation of the brain, followed by the extraction of cortical and subcortical measures. Then, the results from the statistical comparison are reported in tables and graphs to identify potential prognostic factors. There is also an option for plotting results along time. The whole-brain analysis (right) consists of an exploratory voxel-based morphometry (VBM) approach that results in the visualization of a T-statistic image with an overlay of the affected voxels found in the single subject compared to the control group.

The toolbox allows analysis of neurodegeneration based on comparing a control group and a single subject using two different perspectives (see Figure 5).

The ROI-based analysis perspective is focused on specific regions of interest expected to be pathologically affected. In this case, FreeSurfer utilities allow for the parcellation of the brain into cortical and subcortical regions.

More specifically, the anatomical segmentation ROIs were obtained by processing the T1-weighted images of individual participants using the default parameters of the automated segmentation protocol (aparc + aseg) described previously (“recon-all” see Dale et al., 1999; Fischl et al., 1999), then implemented in FreeSurfer v6.0 software^1^. Briefly, brain images were first normalized into Talairach space, and non-brain tissue was excluded. Then, WM bounders were identified by using second intensity normalization, and left and right hemispheres were separated from the isolated WM tissue. After this, the brain stem and cerebellum were removed. Then, a triangular surface tessellation was generated in each hemisphere to define GM/WM and GM/cerebrospinal fluid surfaces. Finally, cortical regions and subcortical regions were segmented into distinct brain tissues using different atlases. Both intensity and continuity information from the whole brain is used to estimate cortical thickness, where the thickness is calculated as the nearest distance from GM-WM to GM-CSF boundary at each vertex on the tessellated surface (Fischl and Dale, 2000). From this parcellation process, different biomarkers (volume and thickness) are extracted to seek differences between a single subject and a control group. This is done using Z-scores and/or a modified version of the independent samples *t*-test, which accounts for the limited size of the control group and was developed for clinical use and single-case research (Crawford and Howell, 1998). This approach contrasts an individual biomarker and normative values derived from the control sample. Although caution has to be taken to evaluate the results, especially in terms of the number of participants and the normality of the control group, it has been successfully used in several MRI studies to run statistical comparisons between a single-subject structural MRI biomarker and a control group (Gillebert et al., 2014; Tuomiranta et al., 2014; Simó et al., 2015). Specifically, the control group is adjusted to the subject’s age by setting a maximum deviation between the age of the subject and the controls’ ages. In this way, the subject is only compared to those controls that are the closest in age. By default, the age deviation is set to 10 (age range of control group = Subject’s age ± 10), but this can be tuned by the user. Although this toolbox only considers age as a confounding factor, it would be also possible to take different potential confounding factors (such as gender, vascular health, years of education, etc.) into account by having a candidate control group matched for the selected factors when asked for selecting the folder that contains controls data.

The false discovery rate (FDR) approach was used to correct all *t*-tests for multiple comparisons based on the number of ROIs tested (*q* = 0.05). Both raw *p*-values (*p*) and the *p*-adjusted FDR values (*p*-adj) are reported. Differences were considered as statistically significant when *p*-adj < 0.05.

SeSBAT offers the option of exploring the longitudinal changes in biomarkers significantly different from the control group by plotting them while controlling for the time between scans. It is therefore necessary to indicate the number of the acquisition session in the input data module.

The whole-brain analysis perspective is a more exploratory analysis, in which no assumptions are made regarding anatomical neural correlates. More specifically, morphometric analysis was carried out based on recommendations for a standardized VBM using the CAT12 toolbox^3^ implemented in the SPM12 software package (Welcome Department of Imaging Neuroscience Group, London, UK) running on MATLAB^®^. To this end, images were realigned, segmented, corrected for signal inhomogeneity, and normalized using the Diffeomorphic Anatomic Registration Through Exponentiated Lie algebra algorithm (DARTEL). Afterward, corresponding normalization parameters were applied to the segmented GM. Subsequently, the resulting GM normalized images were modulated by their Jacobian determinants, allowing direct comparisons of regional differences in GM volume (Mechelli et al., 2005).

At this point, SeSBAT gives the option to plot the resulting registered images using the testing button. Smoothing can also be applied to the images before statistical comparison by selecting the specific kernel. The resulting *T*-statistic image derived from the previously used *T*-test (Crawford and Howell, 1998) can be visualized using the *xjView* toolbox^4^.

## Illustration

In this section, we illustrate the functionality of SeSBAT using a dataset of 42 HD patients, 19 with pre-manifest HD and 23 at the early stages of the disease (I and II, based on total functional capacity, TFC). The normative values to perform the statistical comparison are extracted from a group of 33 healthy controls that were matched with the HD cohort for age (*t*_(70)_ = 0.145, *p* = 0.287) and years of education (*t*_(70)_ = −0.852, *p* = 0.460; see Table 1 for demographic details of HD patients and controls at baseline). We did not discuss the single-subject analysis for the entire cohort, but we illustrate the different analysis steps for some specific participants to make the process reproducible by the user.

**Table 1:** Sociodemographic information of HD patients and controls at baseline.

	Pre-Manifest HD	Manifest HD	HD all	Controls
*N*	19	23^*^	42	33
Gender (f/m)	15/4	13/10	28/14	18/15
Age	36.42 ± 9.18	52.39 ± 10.13	45.17 ± 12.52	44.82 ± 10.59
Years of Education	13.53 ± 2.61	10.74 ± 2.90	12.00 ± 3.08	12.60 ± 2.75
CAG	43.68 ± 2.75	43.96 ± 3.23	43.83 ± 3.04	-
TFC	12.76 ± 0.75	11.26 ± 1.96	11.90 ± 1.72	-
Disease burden	284.61 ± 73.65	421.24 ± 121.15	359.43 ± 122.45	-
Motor domain				
UHDRS-TMS	1.78 ± 3.19	22.57 ± 12.57	13.44 ± 14.15	-
Cognitive domain				
Verbal fluency	141.88 ± 168.53	66.5 ± 106.23	99.59 ± 140.43	-
TMT-B-A	175.2 ± 73.30	291.94 ± 62.07	225.95 ± 90.50	-
SMDT	26.78 ± 11.9	48.50 ± 10.37	36.32 ± 15.58	-
Stroop Interference	46.34 ± 112.75	140.15 ± 168.63	89.69 ± 147.16	-

Data presented as mean ± standard deviation. Age and education are given in years. *Two manifest-HD participants did not complete UHDRS-Cognitive score assessment; *N* = 21 for this cell only. HD, Huntington’s Disease; N, number of participants; f, females; m, males; UHDRS, Unified Huntington’s Disease Rating Scale (Huntington Study Group, 1996); TFC, Total Functional Capacity; TMT B-A, Trail Making. Tests B-A; SDMT, Symbol Digit Modality Test.

MRI data were acquired through a 3T whole-body MRI scanner (Siemens Magnetom Trio; Hospital de Clínic, Barcelona), using a 32-channel phased-array head coil. All participants were acquired in the same scanner at both time points using the same acquisition protocol. Specifically, structural images comprised a conventional high-resolution 3D T1 image (magnetization-prepared rapid-acquisition gradient echo sequence), 208 sagittal slices, TR = 1970 ms, TE = 2.34 ms, TI = 1050 ms, flip angle = 9°, FOV = 256 mm, 1 mm isotropic voxel with no gap between slices. These data were collected at baseline with 18 ± 6 months follow-up of all groups [pre-manifest HD (*N* = 13), manifest HD (*N* = 17), and controls (*N* = 24)]. Also, participants were evaluated in the three symptomatic domains (motor, cognitive, and psychiatric) using a battery of clinical scales and questionnaires. The clinical assessment was carried out using the UHDRS, which comprises motor, cognitive and behavioral subscales. The UHDRS total motor score was selected as a measure of motor disability, with higher scores indicating more severe motor impairment. The cognitive domain was assessed with measures of cognitive flexibility [trail making test (TMT) B-A (Tombaugh, 2004)], verbal fluency (phonemic letter fluency test FAS; Butters et al., 1986), inhibitory control (Stroop interference; Golden, 1978), psychomotor speed (symbol digit modalities test, SDMT; Benedict et al., 2017). In this case, higher scores in TMT B-A and Stroop interference indicate worse performance, while for verbal fluency and SDMT poorer performance corresponds to lower scores.

The proposed toolbox allows two different strategies for the pre-processing and analysis of anatomical MRI data at the single-subject level using specific standardized protocols.

**Figure 6:**
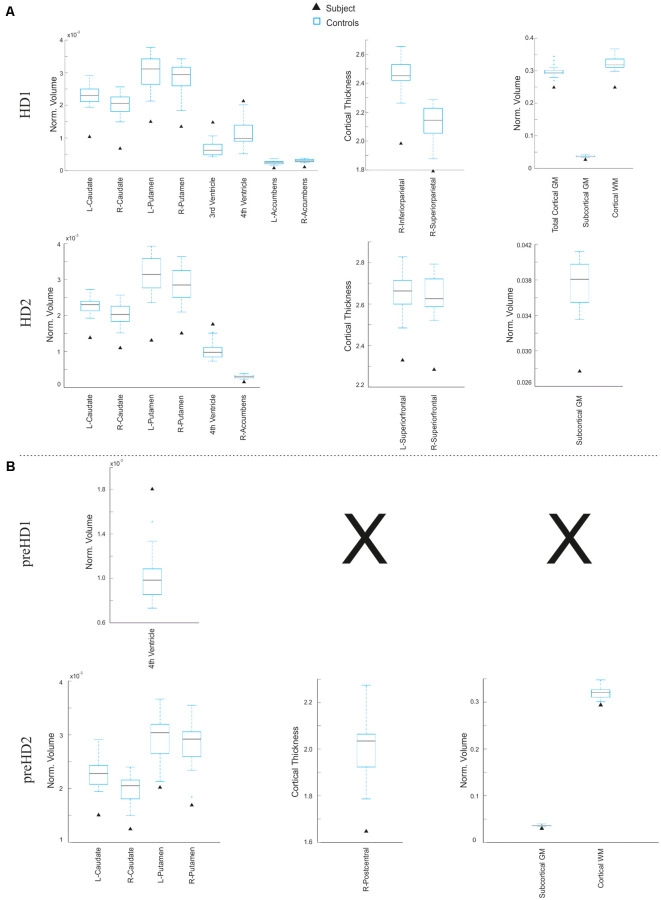
Results derived from the ROI-based analysis. Graphs showing regions in which the subject under study significantly differs from the control group in terms of subcortical volume (left), cortical thickness (center), and global volume of gray matter (GM) and white matter (WM; right) in the manifest **(A)** and pre-manifest **(B)** individuals.

First, in order to illustrate these two types of analysis at subject level, two individual HD patients for each symptomatic stage (pre-manifest and manifest) were randomly selected from the whole sample. We conducted a ROI-based analysis using z-scores as the statistic in the defined target regions of the disease (Figure 6). As expected, both manifest HD patients showed significant cortical thinning and volume loss in several subcortical regions (especially in the caudate, putamen and accumbens area) as well as global subcortical and cortical GM and WM (Figure 6A). In addition, ventricle enlargement was also observed to be significant. when was compared with the corresponding control group. Aligned results were obtained when we correlated the disease burden with the volume of the basal ganglia both in the pre-manifest (R-caudate: *r* = −0.42, *p* = 0.07; L-caudate: *r* = −0.52, *p* = 0.023; R-putamen: *r* = −0.50, *p* = 0.03; L-putamen: *r* = −0.61, *p* = 0.006) and manifest HD groups (R-caudate: *r* = −0.45, *p* = 0.031; L-caudate: *r* = −0.32, *p* = 0.134; R-putamen: *r* = −0.52, *p* = 0.011; L-putamen: *r* = −0.43, *p* = 0.038), and when all HD patients were considered together (R-caudate: *r* = −0.64, *p* = 0.001; L-caudate: *r* = −0.62, *p* = 0.001; R-putamen: *r* = −0.67, *p* = 0.001; L-putamen: *r* = −0.67, *p* = 0.001).

**Figure 7:**
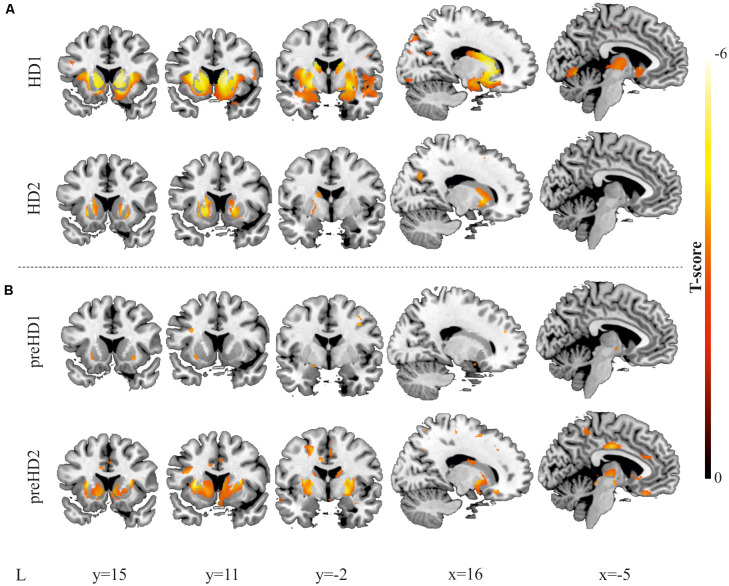
Results derived from the VBM. Decreases in GM are displayed for two individuals with manifest HD **(A)** and two individuals with pre-manifest HD **(B)**. All images are displayed with a significance threshold of *P* < 0.005 (uncorrected). T-scores are indicated by color temperature. The numbers below panels indicate MNI coordinates.

In contrast, larger variability in the affected regions was observed within the pre-manifest patients (Figure 6B), showing no significant anatomical differences with the control group in most biomarkers. In this regard, there is a large body of evidence showing that despite the monogenic nature of HD, there is a high degree of heterogeneity in the prominence and evolution of the clinical symptoms of the pre-manifest phase (Garcia-Gorro et al., 2017). One possible source of such interindividual differences among HD patients could be the variability in the degree of neurodegeneration. For example, the preHD2 patient, displayed in Figure 7, showed a pronounced neurodegeneration pattern compared to the other pre-symptomatic patients, but in the subsequent follow-up assessment, 18 months later became symptomatic. In this line, correlation analyses using the extracted structural biomarkers allow characterizing individual differences in the neurodegeneration pattern corresponding to the clinical symptoms.

Furthermore, VBM analysis was then conducted, and the findings from the comparison with the corresponding control group supported the results from the ROI-based approach. In Figure 7, the higher spatial resolution of the VBM is highlighted, showing larger atrophy in rostral regions of the caudate and the putamen, extending to the insula and the premotor regions. In the pre-symptomatic group, in accord with the results of the ROI-based analysis, higher individual differences between participants were observed, reflecting that the pattern of neurodegeneration is complex at this stage and not always starts or advances following the same trend. To study whether the individual differences of our data can be related to the progression of neurodegeneration, we performed Pearson’s correlation between the number of affected voxels and disease burden.

**Figure 8:**
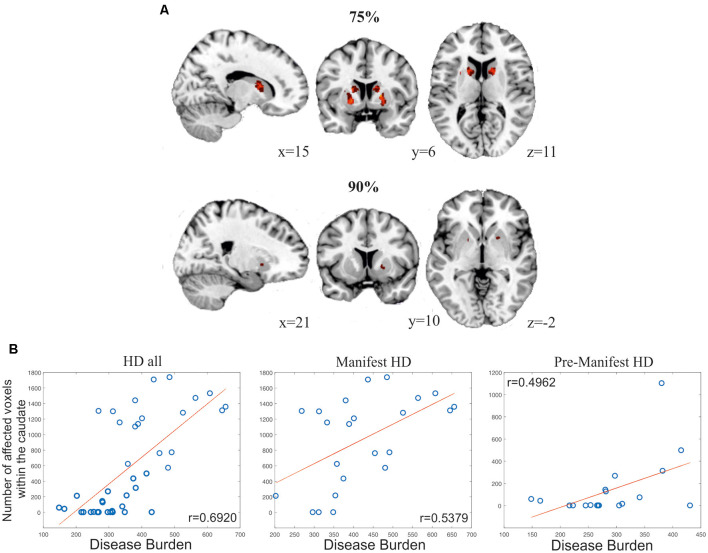
Results from analyses evaluating the global power of SeSBAT for identifying signs of neurodegeneration. **(A)** Overlay of voxels with atrophy that is consistent across 75% (upper panel) and 90% (lower panel) of manifest HD patients. Reported coordinates are MNI coordinates. **(B)** Association between the number of affected voxels within the caudate and disease progression in all HD (left), manifest HD (center), and pre-manifest HD (right) patients. Pearson’s correlation coefficients (r) were reported.

To illustrate the potential of SeSBAT as a tool for uncovering signs of neurodegeneration at the single-subject level, we identified and binarized the results of comparing each manifest HD patient *T*-test with the corresponding control group after thresholding at *p* < 0.005 uncorrected. Finally, all the binary neurodegeneration patterns were added together and normalized to create a probability overlap map of atrophy. Figure 8A illustrates the voxels with atrophy that are consistent across subjects with manifest HD, using two different thresholds (75% and 90%). The reported pattern mirrored the typical striatal neurodegenerative pattern in HD (Harris et al., 1999; Aylward et al., 2004; Paulsen et al., 2008; Hobbs et al., 2010).

The variability in pre-manifest HD groups was larger, and the results did not overlap between subjects at the selected thresholds. Following the previous approach, we created a bilateral mask of the caudate and putamen and computed the percentage of individuals that showed atrophy within those regions. In the selected regions, each voxel was separately analyzed to find the presence or absence of atrophy and then added together. This approach revealed atrophy in the caudate and putamen in 57.89% and 84.21% of pre-manifest HD, respectively, and atrophy in either the caudate or putamen in 89.47% of cases. For the manifest HD group, those values increased to 86.96% and 91.30% for atrophy in the caudate and putamen, respectively (Figure 8B), a biomarker of disease progression that is computed as: Disease Burden = Age × (CAG −35.5) (Penney et al., 1997; Garcia-Gorro et al., 2019). Importantly, the number of voxels with atrophy within the caudate was found to be positively correlated to the disease burden, both in the pre-manifest (*r* = 0.4962, *p* = 0.031, two-tailed) and manifest HD groups (*r* = 0.5379, *p* = 0.008, two-tailed), and when all HD patients were considered together (*r* = 0.6920, *p* = 3.86·10–7, two-tailed).

Additionally, we also investigated whether the individual differences observed in the different symptomatic domains (motor and cognitive) were associated with the different levels of atrophy in the basal ganglia by controlling for the disease burden. Specifically, in the pre-manifest participants, we showed that those participants with lower performance in the different tasks associated with the cognitive domain presented significant lower volume in the basal ganglia (Verbal Fluency: R-caudate: *r* = 0.70, *p* = 0.003; L-caudate: *r* = 0.56, *p* = 0.02; R-putamen: *r* = 0.56, *p* = 0.02; L-putamen: *r* = 0.41, *p* = 0.11); TMT(B-A): (L-putamen: *r* = 0.57, *p* = 0.02); SMDT: (R-caudate: *r* = 0.70, *p* = 0.003; L-caudate: *r* = 0.56, *p* = 0.02; Stroop Interference: R-caudate: *r* = 0.69, *p* = 0.003; L-caudate: *r* = 0.56, *p* = 0.02; R-putamen: *r* = 0.57, *p* = 0.02; L-putamen: *r* = 0.41, *p* = 0.11). However, in the manifest patients, the variability in the reduction of the volume that we found both in the left caudate and left putamen was only related with higher motor alterations (L-caudate: *r* = −0.59, *p* = 0.011; L-putamen: *r* = −0.50*, p* = 0.036). Notice that when all patients were considered together, those individuals with lower volume in the caudate and the putamen, bilaterally, presented significantly higher motor and cognitive alterations.

Moreover, with the follow-up data, we investigated whether the longitudinal changes in the basal ganglia differed between groups. We found that the rate of atrophy was larger in the right putamen in manifest patients (R-putamen: *t*_(28)_ = −2.3, *p* = 0.03, two-tailed), showing a tendency in premanifest patients (R-putamen: *t*_(37)_ = −2.8, *p* = 0.08, two-tailed) compared with controls. Patients were compared to a control group matched by age to control for confounding age effects.

The robustness of this toolbox has been confirmed since the results from the ROI-based and whole-brain analyses replicate the HD neurodegeneration pattern described by previous studies (Tabrizi et al., 2009, 2011; Hobbs et al., 2010).

## Conclusions

SeSBAT has proved to provide potential biomarkers of neurodegeneration at the single-subject level with a more straightforward, handy, and automatic approach, which could quantify and complement the task of neuroradiologists in disease diagnosis and prognosis. Specifically, the proposed toolbox allowed the quantification of the effects of neurodegeneration in both pre- and symptomatic stages, which could lead to the early recruitment of subjects into clinical trials, and aid in designing preventive therapies that can reduce the upcoming cognitive, motor or psychiatric declines. In this article, the power of SeSBAT was limited to its application in studying HD, highlighting that the great potential of this toolbox lies in the possibility of the early detection of subtle changes in the brain that could be a sign of a brain alteration in the pre-symptomatic phase. In HD, neuroimaging is not required for diagnosis because it is provided by genetic testing, so future studies should address identifying patterns of neurodegeneration in other neurodegenerative disorders. Given the similarities between HD and other more common neurodegenerative disorders such as AD and PD, any finding in HD studies could be relevant for making further progress in discovering therapeutic solutions for other neurodegenerative disorders. Moreover, the importance of performing single-subject studies was noted, as patterns of neurodegeneration were found to differ among individuals. This could pave the way towards more personalized treatments tailored to the specific symptomatic profile of each subject.

Despite its promising results, this study has its limitations. Firstly, the results obtained by the toolbox should not be trusted blindly because the segmentation or the normalization derived from FreeSurfer and SPM for the sample tested could be inadequate or suboptimal. The segmentation and normalization result of the automatic algorithm should always be inspected for quality control. Secondly, larger cohorts of healthy controls are essential to perform statistical comparisons using reliable and standardized normative values as a reference. Thirdly, for cross-validation purposes, it would be interesting to test the toolbox with larger data sets such as Track HD and PREDICT, and with other diseases with less individual variability, in which instantaneous and longitudinal changes could be quantified more robustly.

## Data Availability Statement

The datasets generated for this study are available on request to the corresponding author.

## Ethics Statement

The studies involving human participants were reviewed and approved by Clinical Research Ethics Committee of the Bellvitge University Hospital. The patients/participants provided their written informed consent to participate in this study.

## Author Contributions

EC and AP-G contributed to the design of the study and wrote the first draft of the manuscript. AP-G implemented the two-fold strategy provided by SeSBAT. All authors contributed to the article and approved the submitted version.

## Conflict of Interest

The authors declare that the research was conducted in the absence of any commercial or financial relationships that could be construed as a potential conflict of interest.

## Footnotes

^1^http://surfer.nmr.mgh.harvard.edu/

^2^https://www.fil.ion.ucl.ac.uk/spm/

^3^http://dbm.neuro.uni-jena.de/cat/

^4^http://www.alivelearn.net/xjview
